# A204 THE EARLY POST-DISCHARGE PERIOD IS A VULNERABLE PERIOD FOR VENOUS THROMBOEMBOLISM IN PATIENTS WITH INFLAMMATORY BOWEL DISEASE.

**DOI:** 10.1093/jcag/gwae059.204

**Published:** 2025-02-10

**Authors:** A A Seeraj, J McCurdy, D Siegal, E Kuenzig, R Ghasemi, G Tennakoon

**Affiliations:** University of Ottawa, Ottawa, ON, Canada; University of Ottawa, Ottawa, ON, Canada; The Ottawa Hospital Research Institute, Ottawa, ON, Canada; SickKids Research Institute, Toronto, ON, Canada; University of Ottawa, Ottawa, ON, Canada; University of Toronto, Toronto, ON, Canada

## Abstract

**Background:**

Patients with inflammatory bowel disease (IBD) have an increased risk of venous thromboembolism (VTE). The temporal relationship between VTE and hospitalization is poorly understood.

**Aims:**

To determine the proportion of VTE events that are associated with hospitalization in patients with IBD.

**Methods:**

We performed a retrospective cohort study of individuals with Crohn’s disease or Ulcerative Colitis (UC) who developed a VTE between January 1, 2009 and December 31, 2024 at a Canadian academic institution, the Ottawa Hospital. Patients with IBD and VTE events were identified by ICD-10 codes and confirmed with chart review. VTE events were categorized as ambulatory or hospital-associated (within the hospital admission or 90 days of discharge). To account for diagnostic delay at the time of hospitalization, VTE events within 48 hours of admission were categorized as ambulatory events. We determined demographics, comorbidities, medication exposure, anticoagulation and IBD disease activity at the time of VTE diagnosis. Differences between cohorts was determined using Chi-squared or Fisher’s exact test where appropriate.

**Results:**

We identified 275 IBD patients with a VTE event: mean age (SD) was 55 (15.8),132 (48%) female,168 (62%) CD and 103 (38%) UC. The types of VTE included: 49% pulmonary embolism (PE) ± deep vein thrombosis (DVT), 24% DVT alone, 13% portal vein thrombosis and 14 % other VTE (splenic vein, hepatic vein, superior mesenteric vein, cerebral venous sinus). A total of 150 (55%) VTE events were categorized as hospital associated (33 patients (22%) during hospitalization and 117 patients (78%) within 90 days after hospital discharge). 30% of patients in the ambulatory category and 71% of patients in the hospital-associated category (94% during hospitalization and 30% after hospital discharge) were receiving anticoagulation (prophylaxis or therapeutic) prior to the VTE diagnosis. Patients in the hospital-associated category were significantly more likely to have a current malignancy (p=0.01), a severe flare of UC (p=0.008) and receive corticosteroids (p=0.002). There were no differences between cohorts in age, sex, comorbidities, smoking status and anti-TNF exposure.

**Conclusions:**

Our study demonstrates that a large percentage of VTE events in patients with IBD are hospital-associated with the majority occurring early after hospital discharge. Importantly, only a minority of patients received VTE prophylaxis during this period, suggesting the post-discharge period is a vulnerable time for patients with IBD.

**Funding Agencies:**

None

